# A potential new approach for treating systemic sclerosis: Dedifferentiation of SSc fibroblasts and change in the microenvironment by blocking store-operated Ca^2+^ entry

**DOI:** 10.1371/journal.pone.0213400

**Published:** 2019-03-14

**Authors:** Ching-Ying Wu, Wen-Li Hsu, Ming-Hsien Tsai, Chee-Yin Chai, Chia-Jung Yen, Chu-Huang Chen, Jian-He Lu, Hsin-Su Yu, Tohru Yoshioka

**Affiliations:** 1 Department of Dermatology, Kaohsiung Municipal Ta-Tung Hospital, Kaohsiung Medical University Hospital, Kaohsiung Medical University, Kaohsiung, Taiwan; 2 Graduate Institute of Medicine, College of Medicine, Kaohsiung Medical University, Kaohsiung, Taiwan; 3 Department of Cosmetic Science, Chang Gung University of Science and Technology, Taoyuan, Taiwan; 4 Emerging Compounds Research Center, Department of Environmental Science and Engineering, College of Engineering, National Pingtung University of Science and Technology, Pingtung, Taiwan; 5 Department of Child Care, College of Humanities and Social Sciences, National Pingtung University of Science and Technology, Pingtung, Taiwan; 6 Department of Pathology, Faculty of Medicine, Kaohsiung Medical University, Kaohsiung, Taiwan; 7 Department of Pathology, Kaohsiung Medical University Hospital, Kaohsiung Medical University, Kaohsiung, Taiwan; 8 Center for Lipid Biosciences, Kaohsiung Medical University Hospital, Kaohsiung Medical University, Kaohsiung, Taiwan; 9 Vascular and Medicinal Research, Texas Heart Institute, Houston, TX, United States of America; 10 Department of Dermatology, Kaohsiung Medical University, Kaohsiung, Taiwan; 11 Department of Dermatology, College of Medicine, Kaohsiung Medical University, Kaohsiung, Kaohsiung, Taiwan; 12 Graduate Institute of Clinical Medicine, College of Medicine, Kaohsiung Medical University, Kaohsiung, Taiwan; Waseda University, JAPAN

## Abstract

Transforming growth factor-β (TGF-β) is an important target for treating systemic sclerosis (SSc). However, our study revealed three levels of TGF-β1 expression in SSc patients, indicating that inhibiting TGF-β is not sufficient to treat SSc. A previous clinical trial also displayed disappointing results. Thus, our study attempted to search for a potential novel approach. Ingenuity Pathway Analysis (IPA) indicated that the SSc pathological pathways were closely associated with store-operated Ca^2+^ entry (SOCE)-regulated signals, and SOCE activity was found to be increased in SSc fibroblasts. Further treatment of SSc fibroblasts with SOCE inhibitors, 2APB, and associated calcium channel inhibitors SKF96365, and indomethacin, showed that the SOCE inhibitors selectively decreased fibrosis markers and altered the cell morphology. Consequently, SOCE inhibitors, especially 2APB and indomethacin, caused the dedifferentiation of SSc fibroblasts via cytoskeleton remodeling and altered collagen secretion and restored the cell mobility. We further explained SSc pathogenesis as fibroblast differentiation with SOCE. Treatment with exogenous factors, gelatin-1, FAM20A and human albumin, which were identified from the conditioned medium of SSc fibroblasts, was important for regulating the differentiation of fibroblasts with higher levels of SOCE and α-SMA. Conclusively, to treat SSc, blockage of the increased SOCE activity in SSc induces the dedifferentiation of SSc fibroblasts and simultaneously changes the extracellular matrix (ECM) structure to limit SSc pathogenesis.

## Introduction

Systemic sclerosis (SSc), a severe multisystem autoimmune disease, is characterized by progressive fibrosis that can influence all organs in the body [1]. Previous study indicated the majority of SSc deaths involve pulmonary fibrosis, pulmonary arterial hypertension and cardiac causes [2].However, the low efficacy of immunosuppressive treatments suggest a complicated pathogenesis of fibrosis and unknown mechanisms in SSc. For treating fibrotic diseases, especially SSc, several recent studies focused on transforming growth factor-β (TGF-β) as a potential target for anti-fibrotic therapy because TGF-β is a crucial mediator of fibrosis[3].

The excess TGF-β activity in SSc contributes to overproduction of collagen, a component of the extracellular matrix (ECM) [3]. TGF-β or/and TGF-β-induced collagen overproduction can cause the differentiation of fibroblasts to myofibroblasts, and myofibroblasts also accelerate fibrosis by changing ECM collagen fibers [4]. However, blockage of TGF-β to treat fibrosis still faces critical challenges; monoclonal antibodies targeting TGF-β have been used in a clinical trial with disappointing results to date [5]. There is still no efficient approach for treating fibrotic diseases, especially SSc.

To identify an efficient approach for treating fibrotic diseases, we attempted to search for the key factor that is important in regulating SSc development. In addition, in the process of fibrosis in SSc, fibroblasts play a crucial role in interrupting the balance between the ECM and matrix metalloproteinase; in particular, myofibroblasts, which are differentiated from fibroblasts, can dramatically change the ECM structure. Therefore, our study focused on the mechanism underlying the differentiation of fibroblasts in SSc and evaluated whether disrupting myofibroblast activity can inhibit fibrosis.

## Materials and methods

### Cell culture

SSc-fibroblasts were isolated by skin biopsy (5-mm punch) from cutaneous SSc, the area selected were typically tight and firm in clinical condition, and were confirmed by dermatologist, with clinical diagnosis according to the American College of Rheumatology (ACR) criteria as previously described [6]. To avoid inter-individual biologic variation, the control fibroblasts were obtained from paired normal skin tissue of the same patient, which located in symmetrical part of SSc or surrounding of SSc where the texture were relatively normal and not rigid in contrast to the diseased area. The mean age of patients was 45 y (range, 20–68 y), 55% female. Patients had no treatment by any immunosuppressive or steroid therapy. All skin biopsies from SSc patients (n = 20) in this study were received with approval from the Institutional Review Board/Ethics Committee (IRB), number KMUHIRB-F(II)-20160072 in Kaohsiung Medical University Hospital, and approval signed consent were obtained for patients. The isolated procedure of human dermal fibroblasts as described previously [7].

### Calcium imaging

For fibroblasts or SSc fibroblasts pretreated with 2-aminoethoxydiphenyl borate (2APB, 2 μM, Sigma–Aldrich), SKF96365 (20 μM, Sigma–Aldrich) and indomethacin (1 μM, Sigma–Aldrich) for 24 h, intracellular Ca^2+^ responses were induced by the application of 20 nM thapsigargin (TG, Sigma–Aldrich) as previously described [7]. Before the experiments, cells were loaded with 1 μM Fluo-4-AM (Molecular Probes) at 37°C for 20 minutes and then washed with balanced salt solution buffer (5.4 mM KCl, 5.5 mM D-glucose, 1 mM MgSO_4_, 130 mM NaCl, 20 mM Hepes pH 7.4, and 2 mM CaCl_2_). Intracellular Ca^2+^ concentrations ([Ca^2+^]_i_) were calculated from the ratio of fluorescence intensities in the absence of Ca^2+^ and at saturation, emitted (509 nm) upon excitation with consecutive 3-second pulses of 488-nm light at a resolution of 1376 × 1038 pixels using an Olympus Cell^R IX81 fluorescence microscope (Olympus) equipped with an MT 20 illumination system (Olympus) and UPLanApo 10× objective lens. [Ca^2+^]_i_ was estimated based on a [Ca^2+^] calibration curve created using a Ca^2+^ Calibration Buffer kit (Thermo Fisher Scientific). [Ca^2+^]_i_ was calculated using Fluo-4 excited at 488 nm and imaged at 20°C using the same instrument. Fluo-4 signals were calibrated by measuring the fluorescence intensity from microcuvettes containing 10 mM K_2_-EGTA (pH 7.20) buffered to various Ca^2+^ concentrations. [Ca^2+^]_i_ was calculated using the following formula: [Ca^2+^]free = Kd *(F−Fmin/Fmax−F). Plotting the fluorescence intensity versus [Ca^2+^]_i_ yielded the calibration curve with the formula of: [Ca^2+^]_i_ = Kd *(F−Fmin/Fmax−F), where Kd = 345 nM, F = Fluo-4 intensity, Fmax = 640, and Fmin = 21.7 for Fluo-4.

### LC/MS^E^ analysis and IPA

Pretreatment was performed with or without 2APB, SKF96365 and indomethacin in fibroblasts or SSc fibroblasts. After 72 h, cell lysates and conditioned media were collected to analyze the components by using a Waters Xevo G2 qTof mass spectrometer (Waters) as previously described [8]. After LC/MS^E^ analysis, we screened for significant differences in the expression of SSc fibroblasts compared with the paired adjacent fibroblasts in each group by Progenesis QI for Proteomics (QIP) (Waters). For IPA, we uploaded the molecules obtained from the first screening by QIP and identified the common molecules (intersection) among the three groups. The molecules from the intersection among the three groups were further searched to determine the corresponding signaling pathways and associated diseases.

### Wound healing assay

A wound healing assay was performed using IBIDI Culture-Inserts (IBIDI). In brief, 10^4^ cells were plated in each well and were treated with SOCE inhibitors after attachment to the well. After incubating for 24 h, the culture inserts were removed. The movement of cells into the gap area was captured using an Invitrogen EVOS FL Auto 2 Cell Imaging System (Thermo Fisher Scientific) at the indicated time until the gap area had closed. The images of the gap area from the wound healing assay were then measured using ImageJ software (Fiji).

### Western blot analysis

Western blot analyses were performed to measure the protein expression with antibodies against TGF-β1 (GeneTex), vimentin (Abcam), fibronectin (Abcam), α-SMA (Abcam), α-actin (Sigma–Aldrich), β-tubulin (Santa Cruz Biotechnology) and GAPDH (GeneTex), and the method was previously described [9].

### Immunofluorescence staining

Fixed cells were incubated with primary antibodies against vimentin (Abcam), fibronectin (Abcam), and α-SMA (Abcam) at 4°C overnight. The cells were subsequently incubated with secondary antibody for 1 h and then with 4',6-diamidino-2-phenylindole (DAPI, Thermo Fisher Scientific) for 5 min. Coverslips were inverted and fixed onto glass slides by antifade mounting reagent (Biotium). The fluorescence imaging was performed by a confocal microscope (Olympus FV1000).

### Statistical analysis

GraphPad Prism (La Jolla, CA, USA) was used to generate bar charts; error bars indicate the SDs, unless otherwise noted. All statistical analysis data was analyzed by GraphPad-Prism Analysis software (GraphPad-Prism Software Inc., San Diego, CA. USA). Analysis of variance and Student's t-tests were utilized to compare the difference between groups. P-values of less than 0.05 were considered statistically significant for differences between groups.

## Results

### TGF-β1 in skin was not expressed at an increased level in all SSc patients

**Fig 1:**
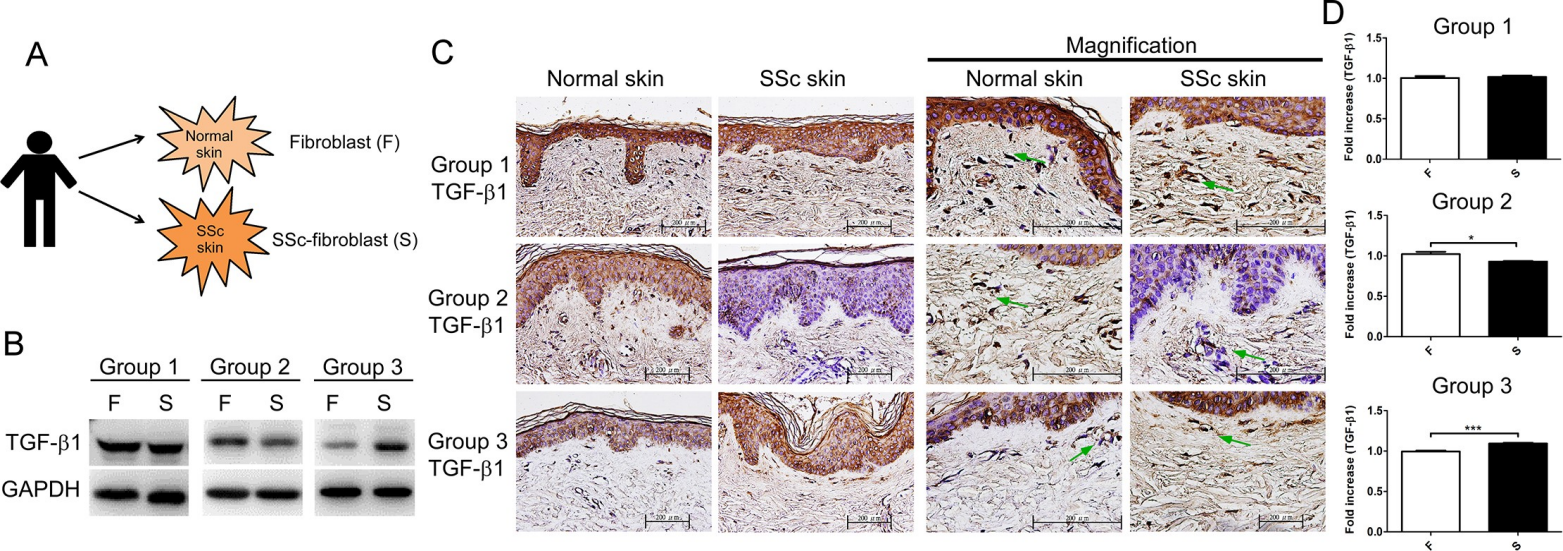
Three levels of TGF-β1 expression were identified in SSc patients. (A) SSc skin tissues and paired adjacent normal skin tissues were collected from these patients according to an Institutional Review Board-approved protocol at Kaohsiung Medical University. Then, SSc fibroblasts (S) and fibroblasts (F) were isolated from SSc skin tissues and normal skin tissues, respectively. (B) Comparison between fibroblasts and SSc fibroblasts showed three types of TGF-β1 expression in these samples: Group 1, no difference between fibroblasts and SSc fibroblasts (N = 4); Group 2, lower level of TGF-β1 expression in SSc fibroblasts (N = 5); and Group 3, higher level of TGF-β1 expression in SSc fibroblasts (N = 11). (C) IHC staining of paraffin-embedded SSc skin tissues and paired adjacent normal skin tissues to show the TGF-β1 expression (brown, counterstained with hematoxylin). Green arrows indicate fibroblasts. (D) Quantification of the TGF-β1 expression as the (C) (mean ± standard deviation (SD), *, *p<0*.*05*; ***, *p<0*.*001*).

Many studies indicated that TGF-β1 is highly expressed in SSc and is a critical target in treating fibrotic diseases [10]. However, our study revealed a completely different result for the expression of TGF-β1 in SSc. We first collected SSc skin tissue and paired normal skin tissue from patients and isolated fibroblasts from the SSc skin and the control skin (Fig 1A). Additionally, the TGF-β1 expression in SSc fibroblasts and control fibroblasts was measured; not all samples displayed a higher level of TGF-β1 than the paired adjacent fibroblasts. Three distinct groups were found by comparing the TGF-β1 expression in the SSc fibroblasts (S) to that in the control group fibroblasts (F): no difference, a lower level and a higher level (Fig 1B). We confirmed the TGF-β1 expression in the SSc skin tissue and the paired skin tissue with immunohistochemical (IHC) staining. The result was similar, as shown in Fig 1C and 1D; interestingly, TGF-β1 was expressed not only in fibroblasts but also in keratinocytes. Our results also indicated three levels of TGF-β1 expression were classified in SSc patients, although increased expression of TGF-β1 in SSc-fibroblasts was revealed with an average of total SSc biopsies (S1 Fig). If keratinocytes of SSc aggravate the activation TGF-β independent to fibroblast [11], extracellular factors may play a certain role in regulation of the microenvironment to induce the differentiation in fibroblasts.

### Store-operated Ca^2+^ entry (SOCE) was activated in SSc fibroblasts and regulated pathological signaling pathways

**Fig 2:**
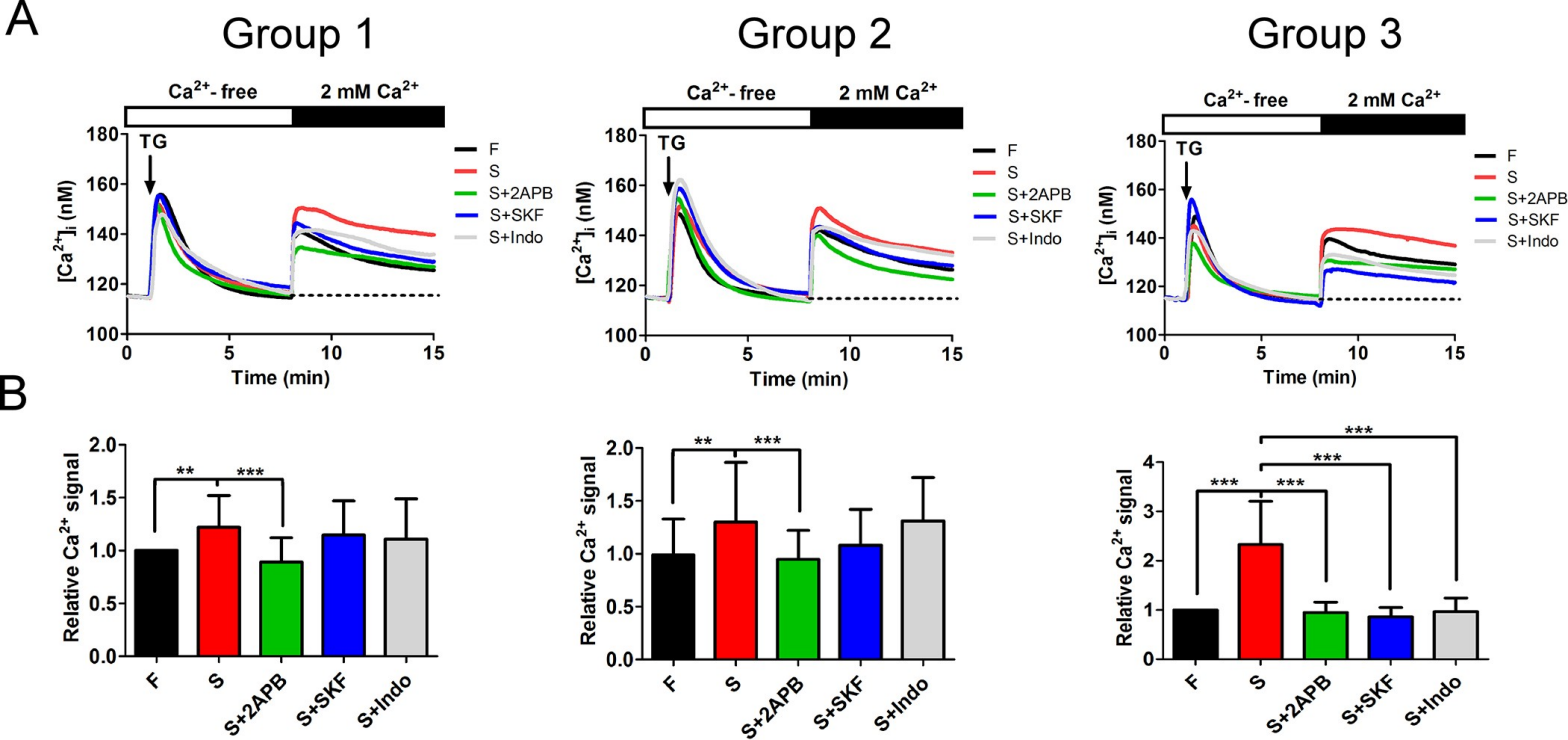
Intracellular Ca^2+^ elevation from SOCE stimulated the pathological signaling pathways in SSc fibroblasts. (A) Comparison of SOCE activity between normal adjacent fibroblasts and SSc fibroblasts in three groups. After application of thapsigargin (TG) (black arrow) to activate store-operated Ca^2+^ channels in Ca^2+^-free balanced salt solution (thick white bar), extracellular CaCl_2_ was added (thick black bar) to induce intracellular Ca^2+^ entry. Treatment with the SOCE inhibitors 2-aminoethoxydiphenyl borate (2APB), SKF96365 (SKF) and indomethacin (Indo). These three inhibitors each significantly limited the TG-induced SOCE activity in SSc fibroblasts. (B) Mean area under the intracellular Ca^2+^ response curves after the addition of extracellular CaCl_2_ in (A) (n >200 cells; mean ± SD, **, *p<0*.*01*; ***, *p<0*.*001*).

TGF-β1 levels are not increased in all SSc tissues, so blocking TGF-β may be insufficient to treat SSc or other fibrotic diseases. Therefore, we determined which factor was critically involved in SSc. As shown in S2A and S2B Fig, 16 molecules are common to the three groups, which are firstly screened by Progenesis QI for Proteomics (QIP), as indicated by Ingenuity Pathway Analysis (IPA). These 16 molecules are associated with increased risk of malignancy in various systemic diseases and disorders (S1 Table). We further classified these 16 molecules by their signaling pathways. The majority of these SSc pathological pathways are closely associated with Ca^2+^ signals, as shown in S2C Fig; store-operated Ca^2+^ entry (SOCE) is the most important Ca^2+^signal associated with inflammation and carcinogenesis [12].SOCE channels include many types of Ca^2+^ channels, such as several transient receptor potential (TRP) channels [13], which are crucial in regulating the proliferation, differentiation, and ECM-protein production in fibroblasts [14]. We found that the SOCE activity was higher in SSc fibroblasts than in the control group fibroblasts by applying thapsigargin (TG) (Fig 2A and 2B). This study also applied three SOCE associated inhibitors, 2-aminoethoxydiphenyl borate (2APB), SKF96365 and indomethacin [6, 9, 15, 16]. Indomethacin as a SOCE associated inhibitor [6, 15] widely used in clinical treatment as an anti-inflammatory agent that provides analgesic effect to SSc patients, and we applied indomethacin in the study for clinical practical concern. Thus, although pretreatment by SKF96365 and indomethacin restrained TG-induced SOCE, the higher SOCE activity in SSc fibroblasts was significantly inhibited by 2APB in the three groups (Fig 2A and 2B). Our results suggested increased SOCE activity is a promising target for treating SSc.

### SOCE inhibitors induced dedifferentiation in SSc fibroblasts

**Fig 3:**
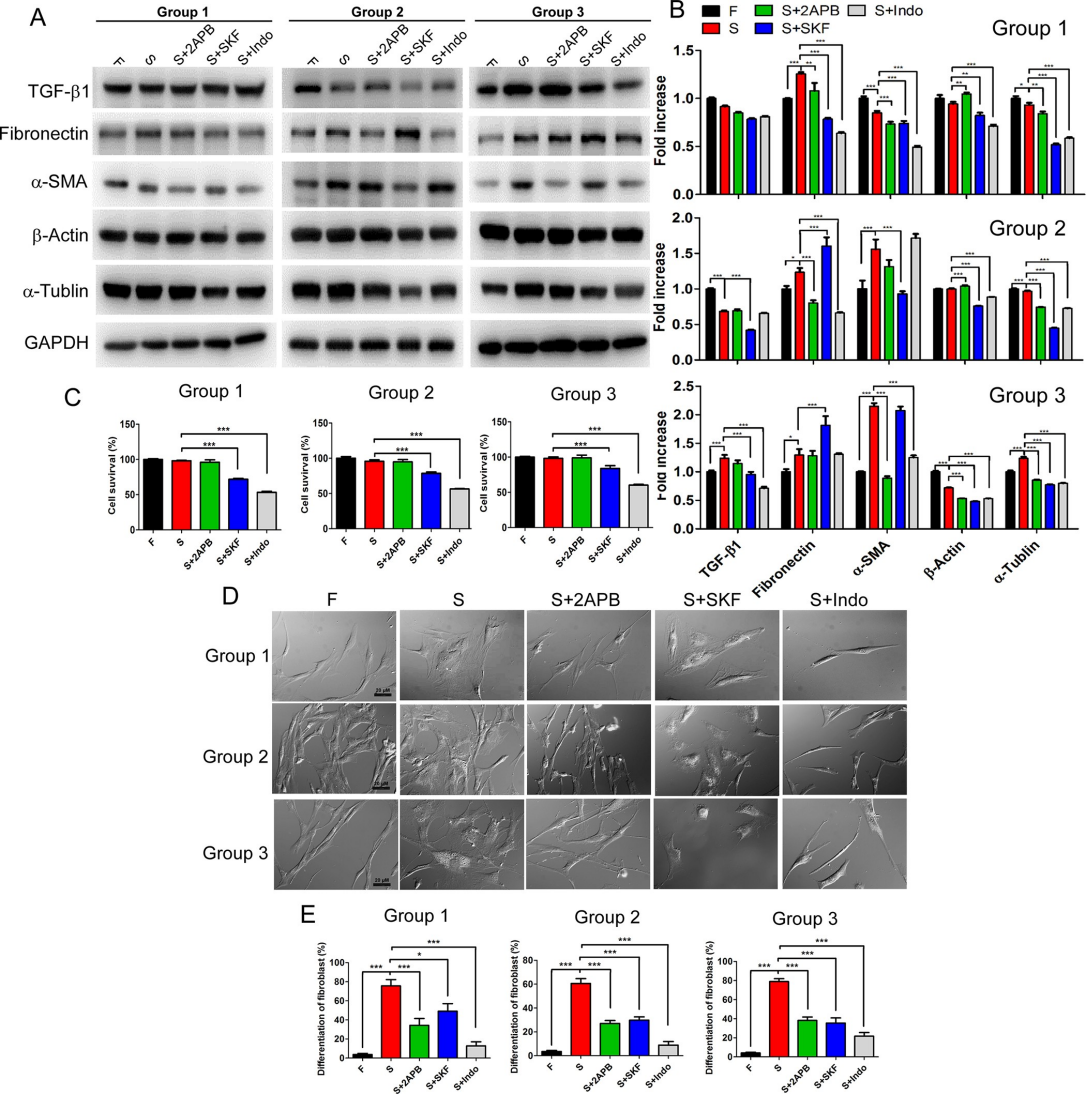
SOCE inhibitors affected fibrosis markers and cell morphology in SSc fibroblasts. After treatment with SOCE inhibitors for 24 h, (A) analysis of the expression of TGF-β1, fibronectin, α-SMA, β-actin, α-tubulin and GAPDH in three groups. The quantification of (A) as the (B) (data were normalized to the protein expression of the internal control, GAPDH; mean ± SD, *, *p<0*.*05*; **, *p<0*.*01*; ***, *p<0*.*001*). (C) Treatment with the SOCE inhibitors SKF and Indo decreased the percentage of cell survival (mean ± SD, ***, *p<0*.*001*). (D) After 72 h, SOCE inhibitors, especially 2APB and Indo, dramatically affected the cell morphology of SSc fibroblasts, indicating dedifferentiation of the SSc fibroblasts. The percentage of differentiated fibroblasts was microscopically quantified in (E), and SOCE inhibitors reduced the percentage of differentiated fibroblasts (mean ± SD, *, *p<0*.*05*; ***, *p<0*.*001*).

Next, we investigated the effect of SOCE inhibitors on the fibrotic pathogenesis in SSc fibroblasts. As shown in Fig 3A and 3B, fibrosis markers, such as TGF-β1, fibronectin and α-SMA, were not consistently expressed at an increased level in SSc fibroblasts in the three groups, although the cell morphology was changed to become similar to myofibroblasts [17], which are differentiated from fibroblasts (Fig 3D). However, treatment with SSc fibroblasts with SOCE and associated inhibitors, 2APB, SKF96365 and indomethacin, led to a selective decrease in fibrosis markers in the three groups (Fig 3A and 3B), mainly the TGF-β1, α-SMA and α-tublin. Although there is discrepancy in the fibronectin and β-Actin expression, in general there is a trend of decreased expression. To note, SKF96365 and indomethacin also influenced the percentage of surviving cells (Fig 3C).

Interestingly, SOCE and associated inhibitors altered the cell morphology of SSc fibroblasts. Fibroblasts are typically bipolar or multipolar, have elongated shapes, and a smooth, regular surface with a large nucleus. In contrast, myofibroblasts, which can be induced by adding TGF-β1, is a more differentiated form of fibroblasts that is much larger and has an undulating membrane and multiple processes. The higher ratio of myofibroblast to fibroblast indicates the extent of fibrosis, in other words, the SSc pathogenesis. Fig 3D revealed that after applying 2APB and indomethacin restored the cell morphology of SSc fibroblasts to that of normal fibroblasts. In order to evaluate whether SOCE inhibitors restored the differentiated fibroblasts through dedifferentiation, we utilized TGF-β1-induced myofibroblasts and treated these cells with SOCE inhibitors for three days. As shown in S3 Fig, the TGF-β1-induced fibroblast differentiation was restored due to dedifferentiation by treatment with SOCE and associated inhibitors, especially 2APB and indomethacin. This finding can be observed by both the morphology of cells and also the expression of the cytoskeletons, mainly α-SMA, which is the well-known cytoskeleton that confined the mobility of myofibroblasts. Furthermore, we also calculated the ratio of differentiated fibroblasts in the three groups through identifying cell morphology, and found that the higher ratio of differentiated fibroblasts in the SSc fibroblast group was significantly decreased by treatment with the SOCE inhibitors (Fig 3D and 3E). Consequently, SOCE and associated inhibitors, especially 2APB and indomethacin, resulted in the dedifferentiation of SSc fibroblasts.

### SOCE inhibitor-induced dedifferentiation of SSc fibroblasts was due to cytoskeleton remodeling and collagen secretion

**Fig 4:**
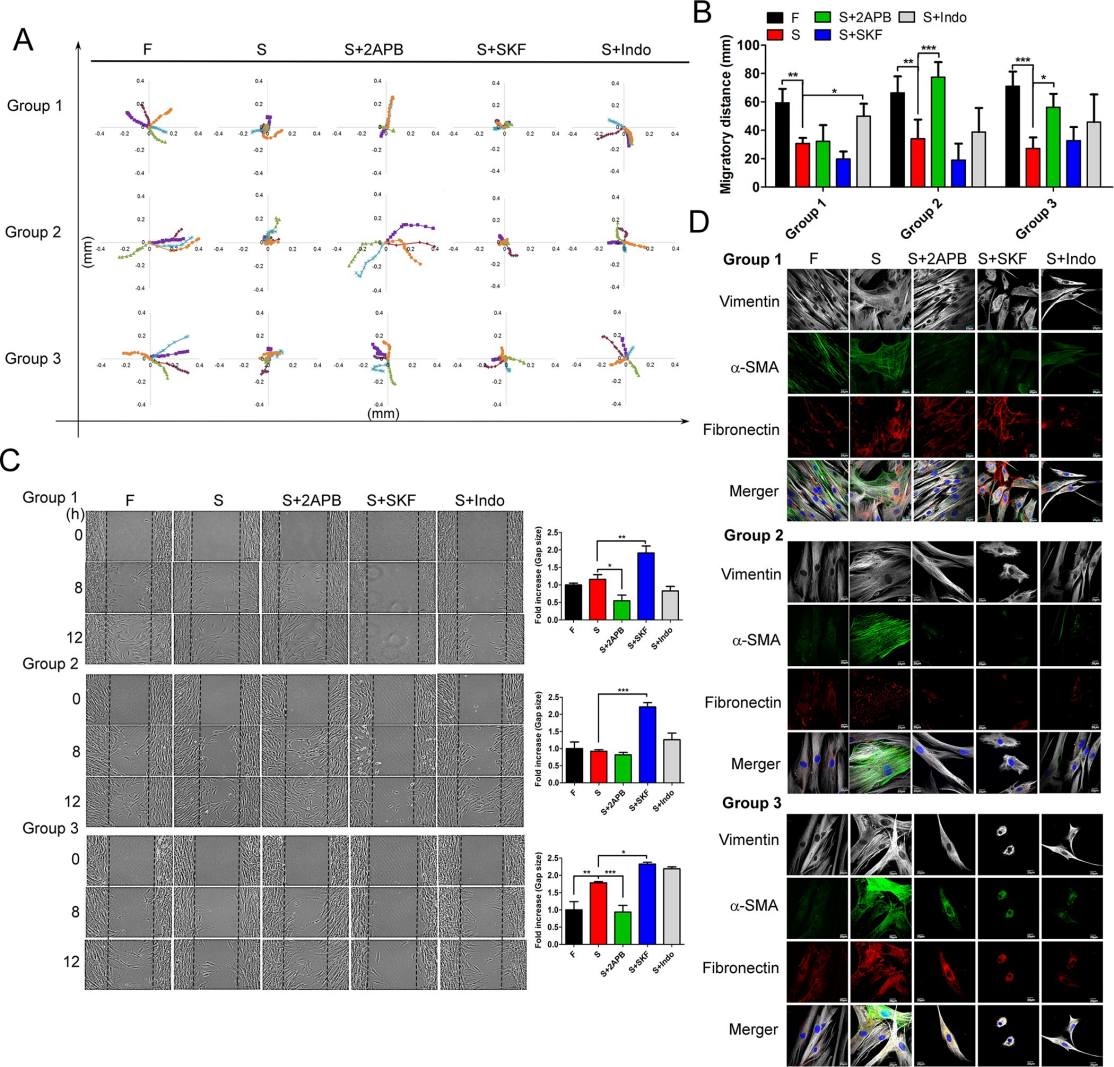
SOCE inhibitors contributed to cytoskeleton remodeling in SSc fibroblasts. (A) Detection of the effect of SOCE inhibitors, as indicated by the cell tracks in the three groups observed under an Olympus Cell^R IX81 fluorescence microscope for 12 h. The X and Y axes shows the migration distance in millimeters (mm). (B) Quantification of migratory distance as the (A) (n >20 cells, mean ± SD, *, *p<0*.*05*; **, *p<0*.*01*; ***, *p<0*.*001*). (C) The migratory ability of three groups was analyzed using a wound healing assay (mean ± SD, *, *p<0*.*05*; **, *p<0*.*01*; ***, *p<0*.*001*). To note, the Y axis showed the gap size, which stands for the empty area without cells. The data shown represent the average of three independent experiments. (D) Analysis of the distribution of vimentin, α-SMA and fibronectin in the three groups; SOCE inhibitors affected the cytoskeleton remodeling in SSc fibroblasts.

Because SOCE inhibitors changed the cell morphology, we supposed that SOCE inhibitors might contribute to dedifferentiation via cytoskeleton remodeling. To prove this hypothesis, we first tracked the motion of cells and found that the SSc fibroblasts migrated in a round circular pattern. However, treatment with 2APB and indomethacin restored the migration pattern of SSc fibroblasts to a pattern similar to that of normal fibroblasts (Fig 4A), and also improved the migratory distance of SSc fibroblasts by calculating the cell migratory trace (Fig 4B). Although the cell migratory capacity cannot be compared between the normal fibroblasts and the three groups of SSc fibroblasts, we found the migratory ability of SSc fibroblasts was increased by 2APB and decreased by SKF96365 (Fig 4C). In brief, 2APB restored the cell mobility of SSc fibroblasts. In addition, the cytoskeleton distribution of SSc fibroblasts was affected by the three types of SOCE inhibitors; the cytoskeleton, as well as the fibrosis markers vimentin, α-SMA and fibronectin, was rearranged and even decreased in response to treatment with SOCE inhibitors (Fig 4D). For instance, the shape and distribution of vimentin fibers reveal straight and tight arrangement in fibroblasts, but the crossed and inattentive arrangement are showed in SSc fibroblasts; treatment by SOCE inhibitors in SSc fibroblasts induced the rearrangement of vimentin fibers, changed the shape and arrangement of vimentin fibers. The similar results are as shown in α-SMA and fibronectin (Fig 4D). Notably, majority of SSc fibroblasts recovered the arrangement of cytoskeletons to fibroblasts by treating with 2APB.

**Fig 5:**
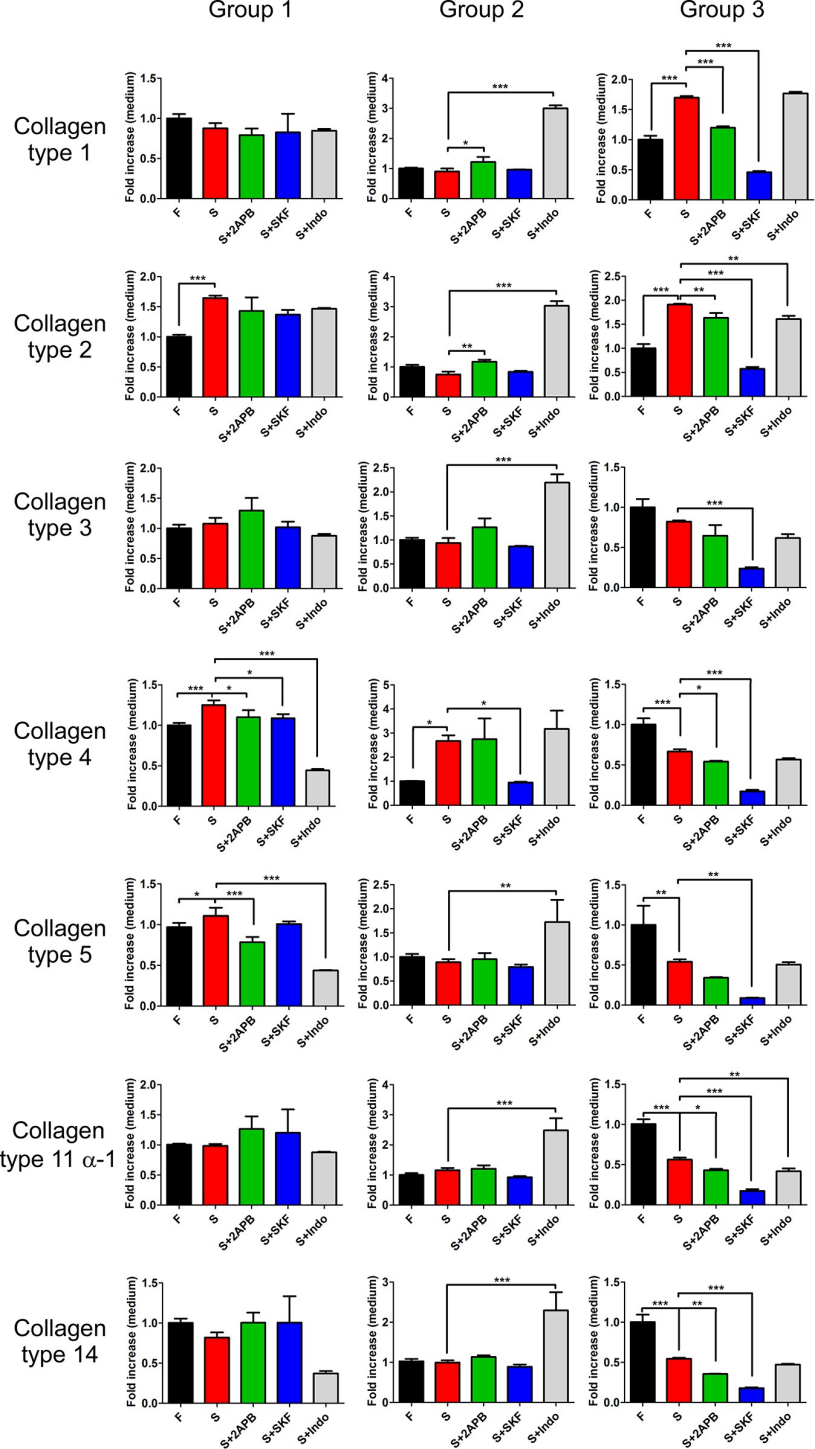
Effect of SOCE inhibitors on the release of different types of collagen from SSc fibroblasts. The level of different types of collagen in the culture medium was altered by treatment with SOCE inhibitors for 72 h (mean ± SD, *, *p<0*.*05*; **, *p<0*.*01*; ***, *p<0*.*001*). The data shown represent the average of three independent experiments.

Furthermore, to consider other changes in addition to cytoskeleton remodeling, we further investigated whether SOCE inhibitors influenced the microenvironment by disrupting collagen secretion. SOCE is important in regulating cytokine release [9].We examined several types of collagen from the medium of SSc fibroblasts: the fibril-forming collagens, collagen types 1,2,3, 5 and 11-α1; the collagen of the basement membrane, collagen type 4; and fibril-associated collagens with interrupted triple helices, type 14 [18]. Our study suggested that SOCE associated inhibitors disrupted the secretion of certain collagens, mainly collagen type 4, and some selective changing in collagen patterns as well as an altered pattern of the ECM microenvironment (Fig 5). Thus, the dedifferentiation of SSc fibroblasts caused by SOCE inhibitors, especially 2APB and indomethacin, was due to cellular cytoskeleton remodeling and an altered ECM composition.

### Exogenous factors were important in regulating the differentiation of fibroblasts with SOCE

**Fig 6:**
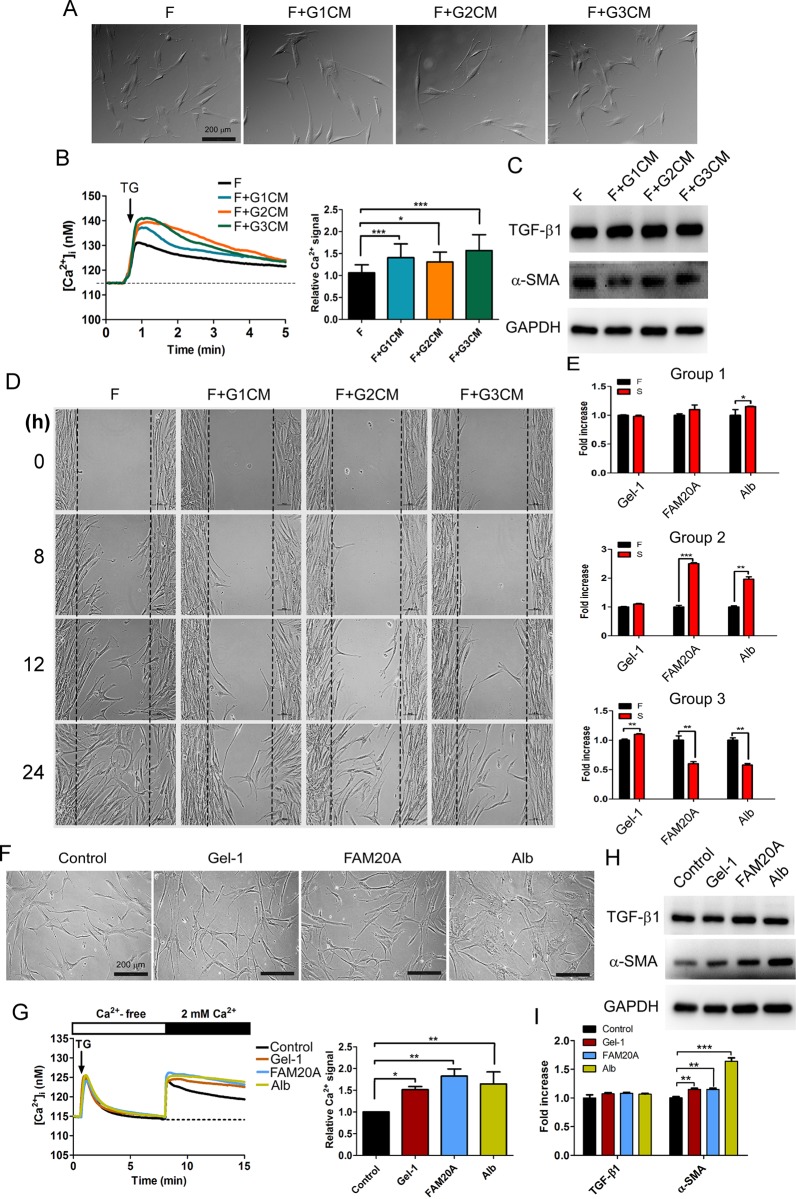
The differentiation of fibroblasts induced by exogenous factors was TGF-β1 independent. (A) Human dermal fibroblasts (F) from healthy people were treated with the conditioned medium of each group, and the alteration in cell morphology was then observed. The conditioned medium was obtained from each group of SSc fibroblasts after a one-day incubation and used to treat the fibroblasts (group 1 conditioned medium, G1CM; group 2 conditioned medium, G2CM; group 3 conditioned medium, G3CM). (B) Treatment with the conditioned medium from each group of SSc fibroblasts promoted the TG-induced Ca^2+^ influx. Right panel: the mean area under the intracellular Ca^2+^ response curves after TG was applied (n >120 cells, mean ± SD, *, *p<0*.*05*; ***, *p<0*.*001*). (C) Analysis of the expression levels of TGF-β1, α-SMA, and GAPDH after treatment with the conditioned medium for 24 h. (D) The conditioned media blocked the cell migration. (E) Three extracellular molecules, gelatin-1 (Gel-1), FAM20A, and human albumin (Alb), were screened by analysis with the IPA system, which identified the common components in the media from the three groups. Analysis of the expression of gelatin-1, FAM20A, and human albumin in the three groups by LC/MS (mean ± SD, *, *p<0*.*05*; **, *p<0*.*01*; ***, *p<0*.*001*). Treatment with gelatin-1 (0.25 ng/ml), FAM20A (1 μg/ml), and human albumin (2 mg/ml) in fibroblasts for 14 days and the subsequent effects on (F) cell morphology, (G) SOCE activity and (H) α-SMA expression. Right panel: the mean area under the intracellular Ca^2+^ response curves after the addition of extracellular CaCl_2_ (n >120 cells, mean ± SD, *, *p<0*.*05*; **, *p<0*.*01*). (I) The quantification of (H) protein expression is shown (data were normalized to the protein expression of the internal control, GAPDH; mean ± SD, **, *p<0*.*01*; ***, *p<0*.*001*).

We further attempted to explain SSc pathogenesis in terms of fibroblast differentiation and SOCE. Previously, we showed that extracellular factors may regulate the microenvironment to induce fibroblast differentiation. Conditioned medium was collected from three groups and used to treat human dermal fibroblasts from healthy people. There was no alteration in cell morphology or in the expression levels of cytoskeletal components and TGF-β1 in response to treatment with the conditional media (Fig 6A and 6C). Notably, the SOCE activity increased, and migratory ability decreased, as shown in Fig 6B and 6D. The condition medium could contain “something” that controlled the SOCE activity, cytoskeletal component expression and migratory ability. We further identified the extracellular factors that were present in the conditioned medium using the IPA system. In the screening of the three groups, gelatin-1, FAM20A and human albumin were the factors identified to be expressed at significantly higher or lower levels among the three groups (Fig 6E). Treatment with gelatin-1, FAM20A and human albumin promoted the expression of the fibrosis marker α-SMA and induced higher SOCE activity, which regulated fibrotic pathogenesis (Fig 6G, 6H and 6I). Treatment with human albumin obviously changed the cell shape with a differentiation from fibroblasts in a TGF-β1-independent process (Fig 6F, 6H and 6I), and restrained the cell migratory ability (S4 Fig). Our results indicated the differentiation of fibroblasts was dependent on extracellular signals, implying that a microenvironmental effect principally contributed to SSc pathogenesis.

## Discussion

In this study we attempted to look for a potential novel approach that would be effective for SSc therapy. Although TGF-β1 is a crucial target in treating SSc, skin biopsies from SSc patients revealed that the TGF-β1 level differed from that of the paired normal skin biopsies. Consequently, based on IPA analysis, we suggested that SOCE inhibitors efficiently attenuated SSc pathogenesis, inducing the dedifferentiation of SSc fibroblasts by cytoskeleton remodeling and changes in collagen secretion. The cytoskeleton remodeling in SSc fibroblasts was due to selective inhibition of cytoskeleton components by SOCE inhibitors; the changes in collagen secretion contributed to disruption of the ECM structure and affected the microenvironmental signals applied to the cells. The microenvironment is the major factor resulting in SSc pathogenesis, and treatment with extracellular factors, such as human albumin, caused fibroblast differentiation. Thus, to treat SSc, endogenous blockage of excess Ca^2+^ signals and exogenous disruption of the ECM structure have potential as novel approaches.

Our study indicated that blocking the increased SOCE activity is an efficient method for treating SSc. SOCE is closely related to the regulation of voltage-gated Ca^2+^ channels (VGCCs) [19].A previous report pointed out that a VGCC inhibitor, nifedipine, disrupted Ca^2+^ signals in fibroblasts and inhibited pulmonary fibrosis in a bleomycin model [20].It is still unclear whether SOCE cooperates with VGCCs, but there is some possibility that inositol triphosphate (IP3; to activate SOCE) and Ca^2+^ (entry by VGCCs) cause Ca^2+^ oscillations through positive feedback [21]. A cell undergoing Ca^2+^ oscillations usually shows increased cellular activity [22]. Nevertheless, VGCCs are known to be an essential players in controlling many physiological functions in excitable cells [23]; the role of VGCCs may be more important in excitable cells than in dermal fibroblasts. The relationship between SOCE and VGCCs in SSc fibroblasts should be further clarified in the future. In addition, may studies mentioned that SOCE is mediated by STIM1-ORAI1[24], but our results revealed that the expression of STIM1 or ORAI1 did not change significantly (S5 Fig). Therefore we will propose here that the increased SOCE activity in SSc fibroblasts might be due to TRP channels, which are potential targets for treating SSc. We further identified the expression of TRPC channels, which are important in regulating SOCE in three groups of SSc, and found that the increased level of TRPC1, TRPC6 and TRPC7.

Interestingly, 2APB, a SOCE inhibitor used in our study, is a powerful candidate for treating SSc because of its low less toxicity and its efficiency in restoring cell mobility. Treatment with 2APB also significantly attenuated the secretion of multiple types of collagen, as shown in Fig 5. Additionally, a nonsteroidal anti-inflammatory drug (NSAID), indomethacin, is frequently utilized in improving the pain of SSc in clinic, but indomethacin has higher toxicity than 2APB (Fig 3C). Actually, treatment with indomethacin caused SSc fibroblasts to recover the normal morphology of fibroblasts, but we found that several cells had become thinner than normal fibroblasts, indicating a change in cell morphology. It is still unclear whether the toxicity of indomethacin contributes to the change in cell morphology. However, the toxicity of SKF96365 disrupts the cytoskeleton, resulting in a decrease in cell mobility (Fig 4D).

According to our study, the pathogenesis of SSc was dependent on direct exogenous effects, such as changes in ECM structure or extracellular molecules; accordingly, changing the microenvironment might be more efficient than targeting fibrosis markers to treat SSc. The fibrosis markers in SSc fibroblasts display different levels of expression; for instance, although we classified the expression of TGF-β1 into three groups, the expression of downstream molecules in TGF-β cascades did not change in a manner consistent with the TGF-β expression in the three groups. This finding that inhibiting the TGF-β1pathway to treat SSc is not sufficiently effective so far. Overall, our study supports the proposal of a new approach for treating SSc: blockage of the increased SOCE activity in SSc induces the dedifferentiation of SSc fibroblasts to restore the cell motility through cytoskeleton remodeling and simultaneously disrupts the ECM structure to limit SSc pathogenesis.

## Supporting information

S1 Fig Increased level of TGF-β1 in SSc-fibroblasts generally. IHC staining of paraffin-embedded SSc skin tissues and paired adjacent normal skin tissues expressing TGF-β1. Quantification of expression of TGF-β1 in fibroblasts (F) and SSc-fibroblasts (S) without classifying (N = 20, mean ± SD, *, *p<0*.*05*).
(DOCX)

S2 Fig The pathological pathways of three groups in SSs-fibroblasts were analyzed by proteomics and bioinformatics approaches. (A) The molecules in each group which were identified through LC/MS, were significantly higher or lower expressed in SSc-fibroblasts when compared with the paired adjacent fibroblasts by Progenesis QI for Proteomics (QIP). (B) These molecules in three groups were analyzed by Ingenuity Pathway Analysis (IPA) comparison, and intersected in specific 16 molecules, which were close relative to SSc pathogenesis. (C) Further analysis performed by using the IPA system revealed the SSc pathological signaling pathways for these 16 molecules. The SSc pathological pathways are regulated via an excess Ca^2+^ signal. The black line or dotted line respectively indicates that the Ca^2+^signal directly or indirectly affects the pathway [25–33].
(DOCX)

S3 Fig SOCE inhibitors induced TGF-β1-induced myofibroblast dedifferentiation. Pretreatment with TGF-β1 (10 ng/ml) to cause human dermal fibroblast differentiation. (A) After treatment with TGF-β1 for three days, the cells differentiated from fibroblasts to myofibroblasts, which were induced to dedifferentiate by treatment with SOCE inhibitors, 2-aminoethoxydiphenyl borate (2APB), SKF96365 (SKF) and indomethacin (Indo). (B) SOCE inhibitors attenuated the expression of fibrosis markers.
(DOCX)

S4 Fig Exogenous factors inhibited the cell migration. (A) Treatment with gelatin-1 (Gel-1), FAM20A, and human albumin (Alb) in fibroblasts for 14 days and the subsequent effect on cell migration. The migratory ability was analyzed using a transwell migration assay kit (Corning Costar), staining the cells which migrated from the top chamber to the lower chamber by crystal violet and counting cells by light microscope. (B) Quantification of the migrated cells as the (A) (mean ± SD, *, *p<0*.*05*; **, *p<0*.*01*; ***, *p<0*.*001*).
(DOCX)

S5 Fig The expression of STIM1 or ORAI1 did not change significantly in SSc fibroblasts. (A) Western blot analysis showing the expression of STIM1, Orai1, and GAPDH. The quantification of (B) STIM1 and (C) Orai1 protein expression is shown (data were normalized to the protein expression of the internal control, GAPDH).
(DOCX)

S1 Table Sixteen molecules from the intersection among the three groups were significantly associated with systemic diseases and disorders through IPA analysis. 16 molecules: annexin A5 (ANXA5), cyclic nucleotide-gated cation channel beta-1 (CNGB1), desmin (DES), fucose-1-phosphate guanylyltransferase (FPGT), glutamate ionotropic receptor AMPA type subunit 4 (GRIA4), IQ domain-containing protein K (IQCK), lactate dehydrogenase A (LDHA), protein disulfide-isomerase A3 (PDIA3), profilin 1 (PFN1), sterile alpha motif domain containing 4B (SAMD4B), SUN domain-containing ossification factor (SUCO), transgelin (TAGLN), class 3 beta tubulin (TUBB3), uveal autoantigen with coiled-coil domains and ankyrin repeats (UACA), vimentin (VIM), and von Willebrand factor A domain-containing protein 3B (VWA3B).
(DOCX)

## References

[1] O'Reilly S. Innate immunity in systemic sclerosis pathogenesis. Clin Sci (Lond). 2014;126(5):329–37. 10.1042/CS20130367 24219159

[2] Tyndall AJ, Bannert B, Vonk M, Airo P, Cozzi F, Carreira PE, et al Causes and risk factors for death in systemic sclerosis: a study from the EULAR Scleroderma Trials and Research (EUSTAR) database. Ann Rheum Dis. 2010;69(10):1809–15. 10.1136/ard.2009.114264 20551155

[3] Varga J, Pasche B. Transforming growth factor beta as a therapeutic target in systemic sclerosis. Nature reviews Rheumatology. 2009;5(4):200–6. 10.1038/nrrheum.2009.26 19337284 PMC3959159

[4] Chia HN, Vigen M, Kasko AM. Effect of substrate stiffness on pulmonary fibroblast activation by TGF-beta. Acta biomaterialia. 2012;8(7):2602–11. 10.1016/j.actbio.2012.03.027 22446029

[5] Varga J, Pasche B. Antitransforming growth factor-beta therapy in fibrosis: recent progress and implications for systemic sclerosis. Current opinion in rheumatology. 2008;20(6):720–8. 10.1097/BOR.0b013e32830e48e8 18946334 PMC4541793

[6] Mo P, Yang S. The store-operated calcium channels in cancer metastasis: from cell migration, invasion to metastatic colonization. Frontiers in bioscience. 2018;23:1241–56. 28930597 10.2741/4641 PMC5632947

[7] Hsu WL, Lu JH, Noda M, Wu CY, Liu JD, Sakakibara M, et al Derinat Protects Skin against Ultraviolet-B (UVB)-Induced Cellular Damage. Molecules. 2015;20(11):20297–311. 10.3390/molecules201119693 26569211 PMC6331914

[8] Wu CY, Hsu WL, Tsai MH, Liang JL, Lu JH, Yen CJ, et al Hydrogen gas protects IP3Rs by reducing disulfide bridges in human keratinocytes under oxidative stress. Sci Rep. 2017;7(1):3606 10.1038/s41598-017-03513-2 28620198 PMC5472599

[9] Wu CY, Hsu WL, Wang CH, Liang JL, Tsai MH, Yen CJ, et al A Novel Strategy for TNF-Alpha Production by 2-APB Induced Downregulated SOCE and Upregulated HSP70 in O. tsutsugamushi-Infected Human Macrophages. PloS one. 2016;11(7):e0159299 10.1371/journal.pone.0159299 27472555 PMC4966960

[10] Pannu J, Gardner H, Shearstone JR, Smith E, Trojanowska M. Increased levels of transforming growth factor beta receptor type I and up-regulation of matrix gene program: A model of scleroderma. Arthritis and rheumatism. 2006;54(9):3011–21. 10.1002/art.22063 16947635

[11] McCoy SS, Reed TJ, Berthier CC, Tsou PS, Liu J, Gudjonsson JE, et al Scleroderma keratinocytes promote fibroblast activation independent of transforming growth factor beta. Rheumatology (Oxford). 2017;56(11):1970–81. 10.1093/rheumatology/kex280 28968684 PMC5850806

[12] Xie J, Pan H, Yao J, Zhou Y, Han W. SOCE and cancer: Recent progress and new perspectives. Int J Cancer. 2016;138(9):2067–77. 10.1002/ijc.29840 26355642 PMC4764496

[13] Earley S, Brayden JE. Transient receptor potential channels in the vasculature. Physiol Rev. 2015;95(2):645–90. 10.1152/physrev.00026.2014 25834234 PMC4551213

[14] Yue Z, Zhang Y, Xie J, Jiang J, Yue L. Transient receptor potential (TRP) channels and cardiac fibrosis. Curr Top Med Chem. 2013;13(3):270–82. 23432060 10.2174/1568026611313030005 PMC3874073

[15] Munoz E, Valero RA, Quintana A, Hoth M, Nunez L, Villalobos C. Nonsteroidal anti-inflammatory drugs inhibit vascular smooth muscle cell proliferation by enabling the Ca2+-dependent inactivation of calcium release-activated calcium/orai channels normally prevented by mitochondria. J Biol Chem. 2011;286(18):16186–96. 10.1074/jbc.M110.198952 21402693 PMC3091227

[16] Tian C, Du L, Zhou Y, Li M. Store-operated CRAC channel inhibitors: opportunities and challenges. Future medicinal chemistry. 2016;8(7):817–32. 10.4155/fmc-2016-0024 27149324 PMC5558521

[17] Sampson N, Berger P, Zenzmaier C. Therapeutic targeting of redox signaling in myofibroblast differentiation and age-related fibrotic disease. Oxid Med Cell Longev. 2012;2012:458276 10.1155/2012/458276 23150749 PMC3486436

[18] Gelse K, Poschl E, Aigner T. Collagens—structure, function, and biosynthesis. Adv Drug Deliv Rev. 2003;55(12):1531–46. 14623400 10.1016/j.addr.2003.08.002

[19] Harraz OF, Altier C. STIM1-mediated bidirectional regulation of Ca(2+) entry through voltage-gated calcium channels (VGCC) and calcium-release activated channels (CRAC). Front Cell Neurosci. 2014;8:43 10.3389/fncel.2014.00043 24605083 PMC3932444

[20] Mukherjee S, Ayaub EA, Murphy J, Lu C, Kolb M, Ask K, et al Disruption of Calcium Signaling in Fibroblasts and Attenuation of Bleomycin-Induced Fibrosis by Nifedipine. American journal of respiratory cell and molecular biology. 2015;53(4):450–8. 10.1165/rcmb.2015-0009OC 25664495

[21] Harootunian AT, Kao JP, Paranjape S, Tsien RY. Generation of calcium oscillations in fibroblasts by positive feedback between calcium and IP3. Science. 1991;251(4989):75–8. 1986413 10.1126/science.1986413

[22] Cheung A, Swann K, Carroll J. The ability to generate normal Ca(2+) transients in response to spermatozoa develops during the final stages of oocyte growth and maturation. Hum Reprod. 2000;15(6):1389–95. 10831575 10.1093/humrep/15.6.1389

[23] Dolphin AC. Voltage-gated calcium channels and their auxiliary subunits: physiology and pathophysiology and pharmacology. J Physiol. 2016;594(19):5369–90. 10.1113/JP272262 27273705 PMC5043047

[24] Cheng KT, Ong HL, Liu X, Ambudkar IS. Contribution and regulation of TRPC channels in store-operated Ca2+ entry. Curr Top Membr. 2013;71:149–79. 10.1016/B978-0-12-407870-3.00007-X 23890115 PMC3824975

[25] Almahbobi G, Korn M, Hall PF. Calcium/calmodulin induces phosphorylation of vimentin and myosin light chain and cell rounding in cultured adrenal cells. Eur J Cell Biol. 1994;63(2):307–15. 8082655

[26] Wang J, Liu J, Cao Y, Hu M, Hua Z. Domain IV of Annexin A5 Is Critical for Binding Calcium and Guarantees Its Maximum Binding to the Phosphatidylserine Membrane. Molecules. 2017;22(12). 10.3390/molecules22122256 29257055 PMC6149819

[27] Trudeau MC, Zagotta WN. Mechanism of calcium/calmodulin inhibition of rod cyclic nucleotide-gated channels. Proc Natl Acad Sci U S A. 2002;99(12):8424–9. 10.1073/pnas.122015999 12048242 PMC123083

[28] Jonas P, Burnashev N. Molecular mechanisms controlling calcium entry through AMPA-type glutamate receptor channels. Neuron. 1995;15(5):987–90. 7576666 10.1016/0896-6273(95)90087-x

[29] Stathopulos PB, Zheng L, Ikura M. Stromal interaction molecule (STIM) 1 and STIM2 calcium sensing regions exhibit distinct unfolding and oligomerization kinetics. J Biol Chem. 2009;284(2):728–32. 10.1074/jbc.C800178200 19019825

[30] Zheng X, Boyer L, Jin M, Mertens J, Kim Y, Ma L, et al Metabolic reprogramming during neuronal differentiation from aerobic glycolysis to neuronal oxidative phosphorylation. Elife. 2016;5 10.7554/eLife.13374 27282387 PMC4963198

[31] Molotkov D, Zobova S, Arcas JM, Khiroug L. Calcium-induced outgrowth of astrocytic peripheral processes requires actin binding by Profilin-1. Cell calcium. 2013;53(5–6):338–48. 10.1016/j.ceca.2013.03.001 23578580

[32] Galligan JJ, Petersen DR. The human protein disulfide isomerase gene family. Hum Genomics. 2012;6:6 10.1186/1479-7364-6-6 23245351 PMC3500226

[33] Lee WH, Kim JY, Kim YS, Song HJ, Song KJ, Song JW, et al Upregulation of class II beta-tubulin expression in differentiating keratinocytes. J Invest Dermatol. 2005;124(2):291–7. 10.1111/j.0022-202X.2004.23506.x 15675945

