# Supplementary figures and images for: A potential new approach for treating systemic sclerosis: Dedifferentiation of SSc fibroblasts and change in the microenvironment by blocking store-operated Ca2+ entry
Source: PLoS One. 2019 Mar 14;14(3):e0213400. doi: 10.1371/journal.pone.0213400 (PMC6417669; doi:10.1371/journal.pone.0213400)

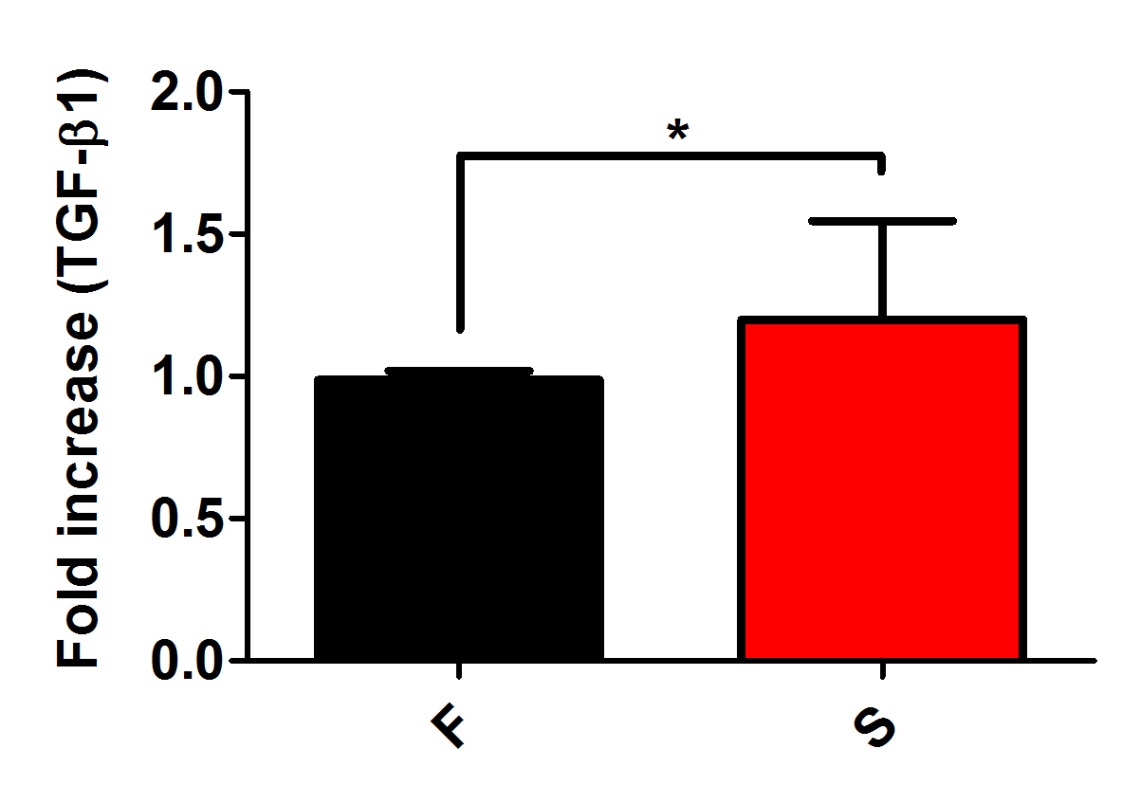


S1 Fig.

Supplement: S1 Fig — IHC staining of paraffin-embedded SSc skin tissues and paired adjacent normal skin tissues expressing TGF-β1. Quantification of expression of TGF-β1 in fibroblasts (F) and SSc-fibroblasts (S) without classifying (N = 20, mean ± SD, *, p<0.05). (DOCX) [file pone.0213400.s001.docx]

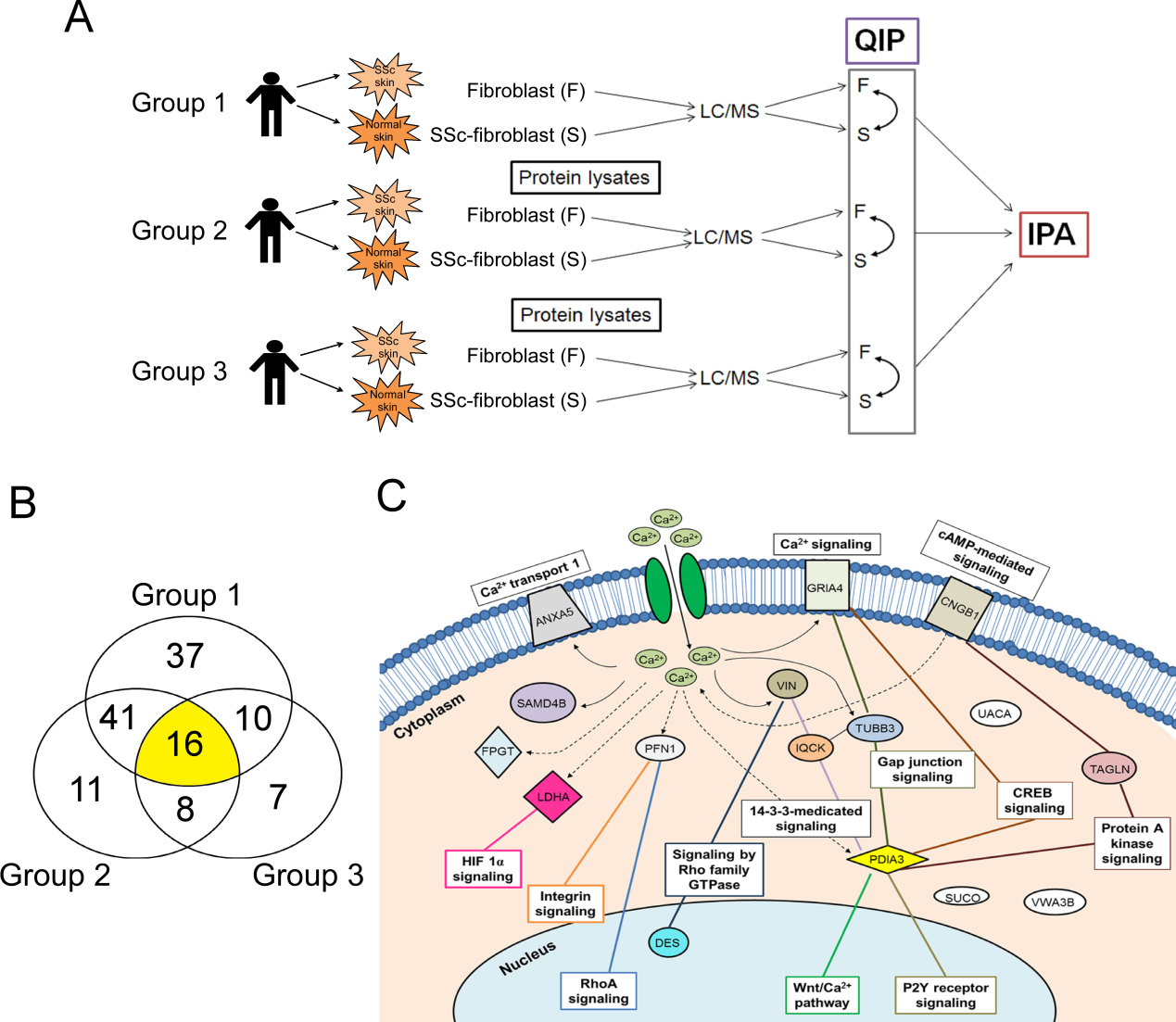


S2 Fig.

Supplement: S2 Fig — (A) The molecules in each group which were identified through LC/MS, were significantly higher or lower expressed in SSc-fibroblasts when compared with the paired adjacent fibroblasts by Progenesis QI for Proteomics (QIP). (B) These molecules in three groups were analyzed by Ingenuity Pathway Analysis (IPA) comparison, and intersected in specific 16 molecules, which were close relative to SSc pathogenesis. (C) Further analysis performed by using the IPA system revealed the SSc pathological signaling pathways for these 16 molecules. The SSc pathological pathways are regulated via an excess Ca2+ signal. The black line or dotted line respectively indicates that the Ca2+signal directly or indirectly affects the pathway [25–33]. (DOCX) [file pone.0213400.s002.docx]

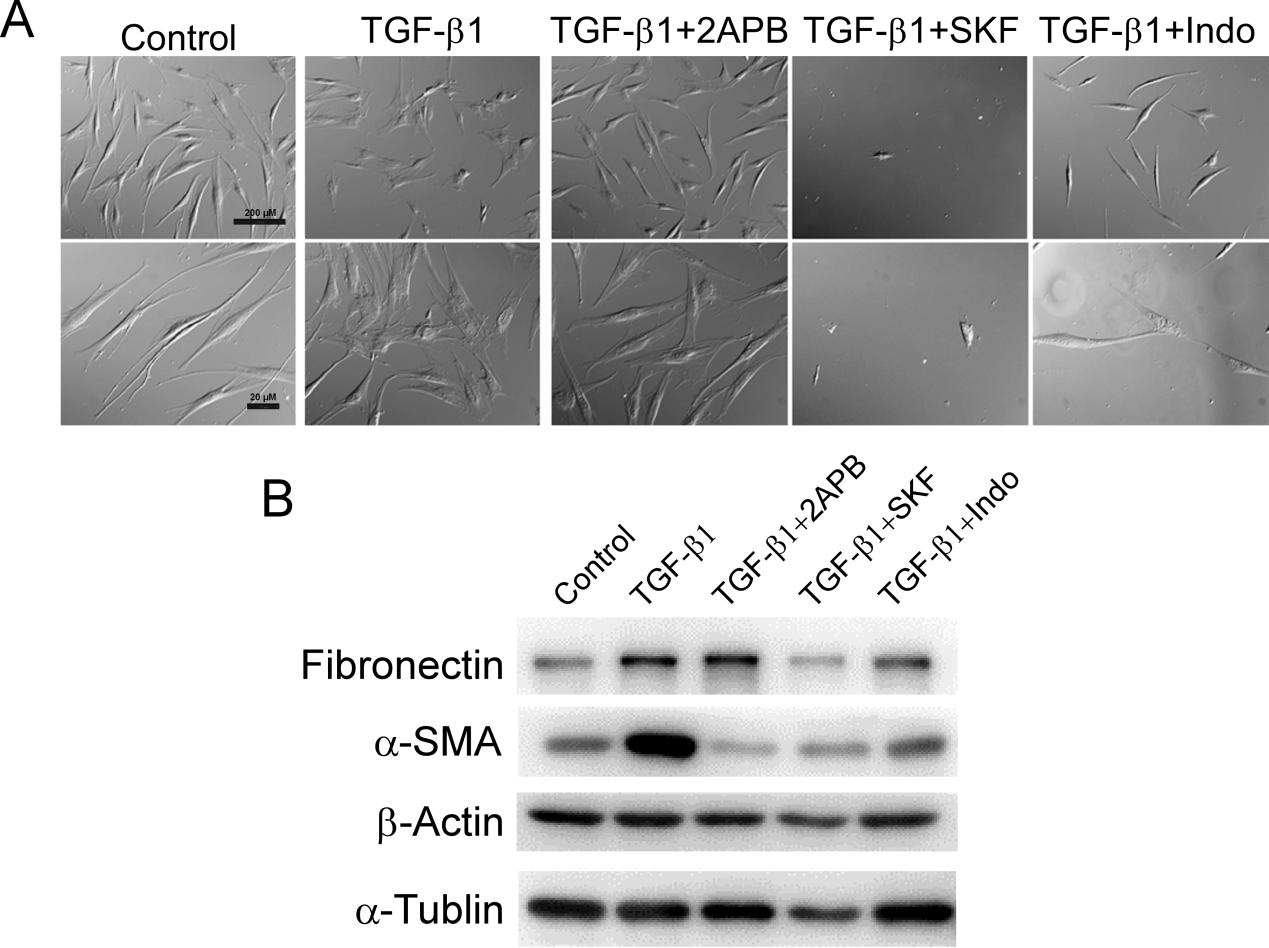


S3 Fig.

Supplement: S3 Fig — Pretreatment with TGF-β1 (10 ng/ml) to cause human dermal fibroblast differentiation. (A) After treatment with TGF-β1 for three days, the cells differentiated from fibroblasts to myofibroblasts, which were induced to dedifferentiate by treatment with SOCE inhibitors, 2-aminoethoxydiphenyl borate (2APB), SKF96365 (SKF) and indomethacin (Indo). (B) SOCE inhibitors attenuated the expression of fibrosis markers. (DOCX) [file pone.0213400.s003.docx]

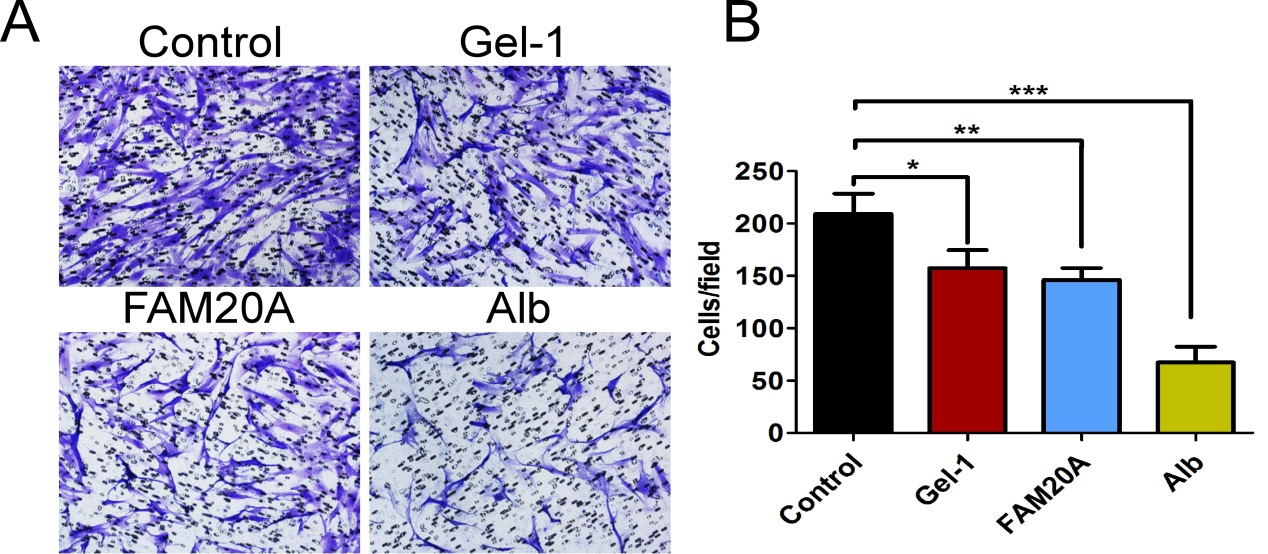


S4 Fig.

Supplement: S4 Fig — (A) Treatment with gelatin-1 (Gel-1), FAM20A, and human albumin (Alb) in fibroblasts for 14 days and the subsequent effect on cell migration. The migratory ability was analyzed using a transwell migration assay kit (Corning Costar), staining the cells which migrated from the top chamber to the lower chamber by crystal violet and counting cells by light microscope. (B) Quantification of the migrated cells as the (A) (mean ± SD, *, p<0.05; **, p<0.01; ***, p<0.001). (DOCX) [file pone.0213400.s004.docx]

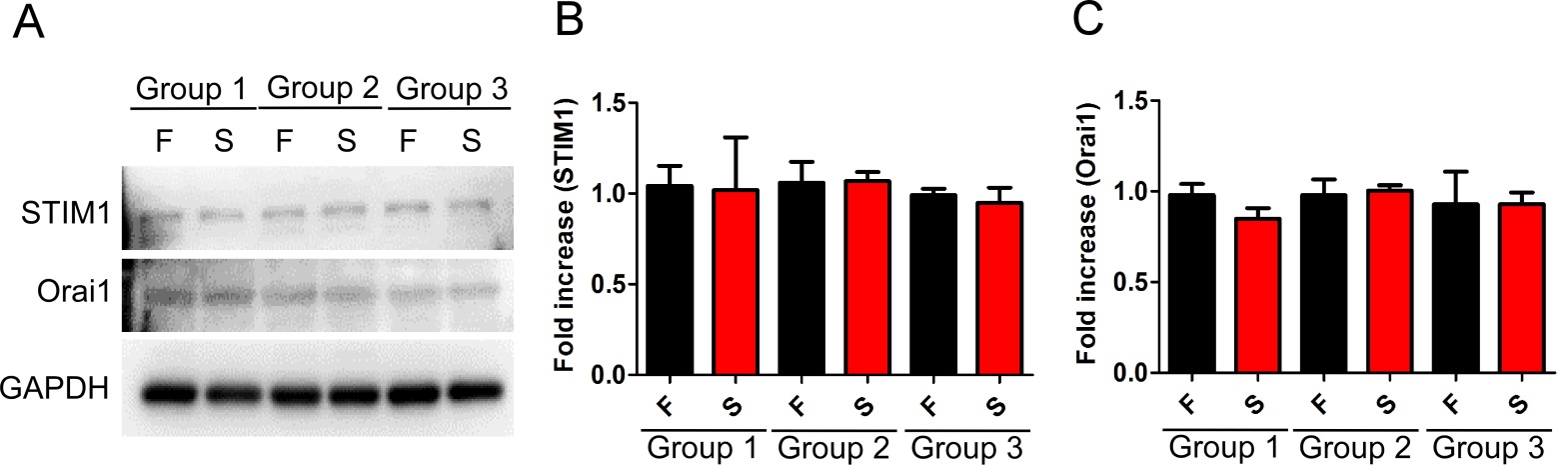


S5 Fig.

Supplement: S5 Fig — (A) Western blot analysis showing the expression of STIM1, Orai1, and GAPDH. The quantification of (B) STIM1 and (C) Orai1 protein expression is shown (data were normalized to the protein expression of the internal control, GAPDH). (DOCX) [file pone.0213400.s005.docx]
