# Supplementary material for: A potential new approach for treating systemic sclerosis: Dedifferentiation of SSc fibroblasts and change in the microenvironment by blocking store-operated Ca2+ entry
Source: PLoS One. 2019 Mar 14;14(3):e0213400. doi: 10.1371/journal.pone.0213400 (PMC6417669; doi:10.1371/journal.pone.0213400)
Supplement: S1 Table — 16 molecules: annexin A5 (ANXA5), cyclic nucleotide-gated cation channel beta-1 (CNGB1), desmin (DES), fucose-1-phosphate guanylyltransferase (FPGT), glutamate ionotropic receptor AMPA type subunit 4 (GRIA4), IQ domain-containing protein K (IQCK), lactate dehydrogenase A (LDHA), protein disulfide-isomerase A3 (PDIA3), profilin 1 (PFN1), sterile alpha motif domain containing 4B (SAMD4B), SUN domain-containing ossification factor (SUCO), transgelin (TAGLN), class 3 beta tubulin (TUBB3), uveal autoantigen with coiled-coil domains and ankyrin repeats (UACA), vimentin (VIM), and von Willebrand factor A domain-containing protein 3B (VWA3B). (DOCX) [file pone.0213400.s006.docx]

| **Categories** | **Disease or Functional Annotations** | **Molecules** |
| --- | --- | --- |
| Cancer | Carcinoma | ANXA5, CNGB1, DES, FPGT, GRIA4, IQCK, LDHA, PDIA3, PFN1, SAMD4B, SUCO, TAGLN, TUBB3, UACA, VIM, VWA3B |
| Neurological Disease | Progressive motor  neuropathy | ANXA5, LDHA, PDIA3, PFN1, VIM |
|  | Movement Disorders | LDHA, PFN1, TUBB3, VIM, VWA3B |
|  | Tauopathy | PDIA3, TAGLN, TUBB3, VIM |
|  | Neuromuscular  disease | LDHA, PDIA3, PFN1, VIM |
|  | Disorder of  basal ganglia | LDHA, PFN1, TUBB3, VIM |
|  | Amyotrophic  lateral sclerosis | ANXA5, PFN1, VIM |
|  | Paralysis | PFN1, TUBB3 |
| Cardiovascular Disease | Atherosclerosis | GRIA4, PFN1, TUBB3, VWA3B |
|  | Peripheral vascular disease | SUCO, TUBB3, VIM |
|  | Adhesion of vascular  endothelial cells | ANXA5, VIM |
|  | Formation of  blood vessels | LDHA, TUBB3 |
|  | Hypertrophy of the heart | DES, PFN1 |
|  | Coronary artery disease | GRIA4, TUBB3, VWA3B |
| Endocrine System Disorders | Experimentally induced  diabetes | ANXA5, CNGB1 |
| Carbohydrate Metabolism | Glycolysis of cells | LDHA, PFN1 |
| Inflammatory Response | Inflammation of lung | ANXA5, PDIA3, UACA |
| Connective Tissue Disorders | Scleroderma | PDIA3, VIM |
| Cellular Movement | Cell movement of breast  cancer cell lines | LDHA, PFN1, VIM |
| Cell-To-Cell Signaling and Interaction | Phagocytosis of cells | ANXA5, PFN1, VIM |
|  | Phagocytosis of tumor  cell lines | PFN1, VIM |
| Cellular Function and Maintenance | Endocytosis by  eukaryotic cells | ANXA5, PFN1, VIM |
| Cellular Assembly and Organization | Organization of  cytoskeleton | DES, PDIA3, PFN1, TUBB3, VIM |
|  | Formation of  intermediate filaments | DES, VIM |
|  | Organization of  intermediate filaments | DES, VIM |
| Protein Synthesis | Polymerization of protein | ANXA5, CNGB1, PFN1 |
| Cell Morphology | Shape change of  breast cancer cell lines | ANXA5, PFN1 |
| Molecular Transport, Protein Trafficking | Import of protein | PDIA3, UACA |
| Development | Formation of muscle | ANXA5, DES, VIM |
|  | Development of  central nervous system cells | SAMD4B, VIM |
|  | Guidance of axons | PDIA3, TUBB3 |
| Organismal Functions | Muscle weakness | DES, PFN1 |

S1 Table.
